# Role of solution concentration in formation kinetics of bromide perovskite thin films during spin-coating monitored by optical *in situ* metrology†

**DOI:** 10.1039/d2ra06314j

**Published:** 2022-11-15

**Authors:** C. Rehermann, V. Schröder, M. Flatken, F. Ünlü, O. Shargaieva, A. Hoell, A. Merdasa, F. Mathies, S. Mathur, E. L. Unger

**Affiliations:** Department of Solution-Processed Materials and Devices, HySPRINT Innovation Lab, Helmholtz-Zentrum Berlin für Materialen und Energie GmbH Kekuléstraße 5 12489 Berlin Germany carolin.rehermann@helmholtz-berlin.de eva.unger@helmholtz-berlin.de; Helmholtz Zentrum für Materialien und Energie GmbH Hahn-Meitner-Platz 1 14109 Berlin Germany; Department Novel Materials and Interfaces for Photovoltaic Solar Cells, HySPRINT Innovation Lab, Helmholtz-Zentrum Berlin für Materialen und Energie GmbH Kekuléstraße 5 12489 Berlin Germany; Inorganic and Materials Chemistry, University of Cologne Greinstr. 6 50939 Cologne Germany; Department of Clinical Sciences Lund, Lund University Sölvegatan 17 Lund Sweden; Hybrid Materials: Formation and Scaling, IRIS Adlershof, Humboldt Universität zu Berlin Am Großen Windkanal 2 12489 Berlin Germany; Chemical Physics and Nano Lund, Lund University Lund Sweden

## Abstract

Optoelectronic devices based on metal halide perovskites continue to show a improved performance, and solution-based coating techniques pave the way for large-area applications. However, not all parameters influencing the thin film formation process of metal halide perovskites are identified and entirely rationalised over their full compositional range, thus hampering optimised thin film fabrication. Furthermore, while the perovskite deposition *via* spin-coating and annealing is an easily accessible technique, more profound insights into the chemical formation process are still lacking. Varying the precursor solution concentration is commonly used to vary the resulting thin film thickness. This study shows that varying the precursor solution concentration also affects the thin film morphology and optoelectronic quality. Hence, we herein investigate the influence of the precursor solution concentration on the formation process of a pure bromide-based triple cation perovskite (Cs_0.05_MA_0.10_FA_0.85_PbBr_3_) by fiber-based optical *in situ* measurement. During the spin-coating process, *in situ* UV-vis and PL measurements reveal formation kinetics are strongly dependent on the concentration. Furthermore, we identify delayed nucleation and retarded growth kinetics for more concentrated precursor solutions. In addition, we quantify the shifting chemical equilibrium of colloidal pre-coordination in the precursor solution depending on concentration. Namely, colloids are pre-organised to a higher degree and higher-coordination lead–bromide complexes tend to form in more concentrated precursor solutions. Thus, the modified solution chemistry rationalises retarded perovskite formation kinetics and highlights the precursor concentration as an influential and optimisable parameter for solution-based thin film deposition.

## Abstract 2

The solution chemistry of the precursor ink defines the formation kinetics of metal halide perovskites. Thus, rationalizing the formation process from solution *via* the deposition paves the way for high-quality thin films for optoelectronic devices.

## Graphical abstract

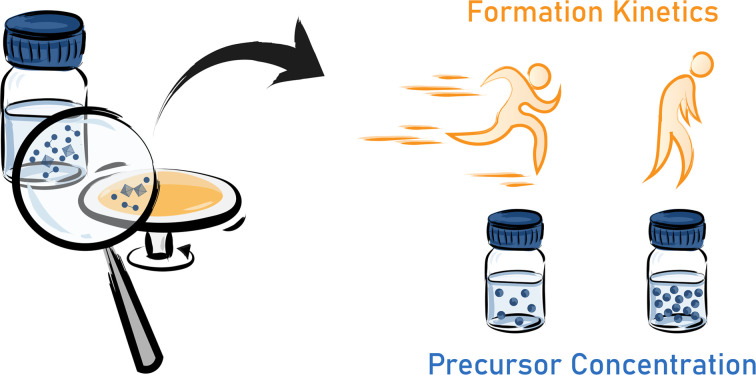

## Introduction

Over the last decade, metal halide perovskites (MHPs) rose to prominence in materials and renewable energy research due to their outstanding optical properties, namely a steep absorption onset and a high absorption coefficient,^1^ as the photoactive material in optoelectronic devices. Perovskite-based single junction and tandem solar cell devices have today reached record efficiencies of 25.7% and 31.3%, respectively.^2^ A unique benefit of MHPs is their deposition *via* solution-based techniques, such as spin-coating in a laboratory and printing or continuous coating techniques on an industrial scale.^3^ Thus, production costs are reduced, and MHPs are brought into the game to compete with or complement established silicon-based solar cells.^4^ In addition, the unique bandgap tunability of MHPs^5^ sparks interest in extending their utilisation into further optoelectronic applications, *e.g.*, light emitting diodes (LEDs),^6^ transistors,^7^ and detectors.^8^

A remaining challenge is the reliable and reproducible fabrication of metal halide perovskite thin films over their manifold compositional range. High-quality thin films are essential for manufacturing and improving perovskite-based optoelectronic devices.^9–11^ Thus, the solution-based perovskite thin film deposition has been focused on optimising the layer quality in morphology and film thickness. Such optimisation is achieved by tailoring, *e.g.*, the solvent system,^12–14^ the precursor and spectator salts,^15–17^ setting an anti-solvent drop,^18^ and adjusting technical preparation parameters.^19–21^ The preparation routine and related formation pathways dictate the thin film quality. Thus, understanding and rationalising perovskite formation processes is key to reliable and reproducible high-quality perovskite thin films. Nevertheless, the formation process's precursor solution chemistry and individual preparation parameters are little explored and understood.

Several intermediate solvate and crystalline phases have been identified, and their occurrence depends on the exact precursor composition and preparation route.^22–27^ Lately, studies have focused on clarifying the formation process of standard perovskite compositions and recipes, such as MAPbI_3_ and (Cs_0.05_MA_0.17_FA_0.83_)Pb(Br_0.17_I_0.83_)_3_ (so-called “triple cation”), by optical and structural *in situ* measurements. Merdasa *et al.*^19^ describe the overall formation process of the “triple cation” perovskite during spin-coating and annealing, and examine wet-film thinning. Sutter-Fella and co-workers identify different formation dynamics for MAPbI_3_ determined by the lead salt in the precursor solution^28^ and an optimised anti-solvent dripping time related to the final MAPbI_3_ thin-film properties.^29^ In addition, Taylor *et al.*^30^ classified three types of anti-solvents and their utilisation, explaining differences in solar cell performance.

Such top-down approaches aim to rationalise established preparation routines and reason the device performance. Evaluating various preparation procedures from “The Perovskite Database Project”^31,32^ reveals that 97% of metal halide perovskite thin films are prepared from solution-based processes, 68% of those are fabricated by conventional 1-step spin-coating, and a further 24% include spin-coating as the primary preparation step. Spin-coating preparation depends on parameters such as spin speed, acceleration and time, temperature, atmosphere, annealing temperature and time, solvent system, anti-solvent, precursor salts, and solution concentration. 286 solvent and 93 anti-solvent combinations are currently utilised in literature^31^ and reflect on the herculean task of optimising thin-film fabrication with empirically determined solvent systems. Thus, a bottom-up approach rationalising the influence of individual preparation parameters will enable targeted solution-based process development, increase the reproducibility, and perovskite thin film quality. The first attempts were made by Merdasa *et al.*^19^ investigating the thinning of the wet film with increasing spin speed and overall crystallisation processes *via* optical *in situ* monitoring. Furthermore, varying the halide ratio to tune the bandgap influences the formation pathways of MHPs. They form directly from solution, *via* an intermediate solvate phase, or *via* both competing pathways depending on the halide ratio.^33^

The precursor solution concentration is one modifiable parameter for perovskite preparation, used to adjust the thin film thickness. Usually, a relatively high concentration is used for solar cell application (∼1.2 M)^34^ with spin-coating techniques. In comparison, lower solution concentrations are practical in printing and LED manufacturing.^6^ Effects of changes in the precursor concentration on the formation kinetic and thin film quality beyond film thickness were not yet discussed. Therefore, we report and discuss the influence of the precursor solution concentration in detail on the formation kinetics of MHPs and underlying solution chemistry, using the example of FA_0.85_MA_0.10_Cs_0.05_PbBr_3_ (3CatPbBr_3_). Kulbak *et al*.^35^ introduced this perovskite composition as the most promising for wide bandgap, stable and high-efficiency solar cell devices. Significant improvements are postulated upon optimisation of the 3CatPbBr_3_ thin-film quality. However, especially wide-bandgap bromide-based perovskites are additionally applicable for LEDs.^6^ LEDs, as all technologies based on thin films, require a high thin film quality. Thus, rationalising underlying film formation processes is essential to optimise and increase the performance of bromide-based MHPs, not only for solar cells but for a broader range of optoelectronic applications. Also, optimising large-scale deposition will enormously benefit from understanding the formation process from solution to the final film and make optimisation more efficient and target-oriented.

Bromide-based perovskites form directly from solution during spin-coating as they interact weaker with solvents^36^ than their iodide-based counterparts. Iodide-based perovskites form *via* an intermediate step.^27^ For this reason, this fundamental study on the influence of the solution concentration on the formation kinetics focuses on bromide-based perovskites and deliberately omits the iodide-based materials. Rationalising formation kinetics is most suitable for bromide-based perovskites since additional intermediate and parallel formation steps can be neglected. Thus, data interpretation and conclusion are streamlined since only the formation from solution onto the expected perovskite needs to be considered.

We observe a drastic impact of the precursor solution concentration on the formation kinetics utilising *in situ* UV-vis and PL spectroscopy. Counterintuitively, an earlier crystallisation onset and a faster crystallite growth during spin-coating are identified for lower precursor solution concentrations. These formation kinetics are unexpected concerning the conventional LaMer model commonly used to describe perovskite crystallisation processes.^37,38^ Higher concentrated solutions should reach super-saturation faster due to solvent evaporation and, thus, crystallise earlier.

Small Angle X-ray Scattering (SAXS) and Nuclear Magnetic Resonance (NMR) measurements on the solution concentration series reveal an increased degree of crystallite pre-order for higher concentrated precursor solutions and the formation of colloids. Thus, complex and colloid solution chemistry changes evoked an earlier crystallisation onset and faster growth kinetics for lower concentrated solutions. The higher the solution concentration, the slower the formation kinetics since preformed colloidal structures need to re-structure and re-organise. Thus, the precursor concentration dictates the solution chemistry and predefines critical factors in the formation process. Overall, this study contributes to a systematic characterisation of perovskite preparation parameters and highlights the importance of underlying precursor solution chemistry and characteristics for perovskite formation processes.

## Results and discussion

### Thin-film characteristics

**Fig. 1:**
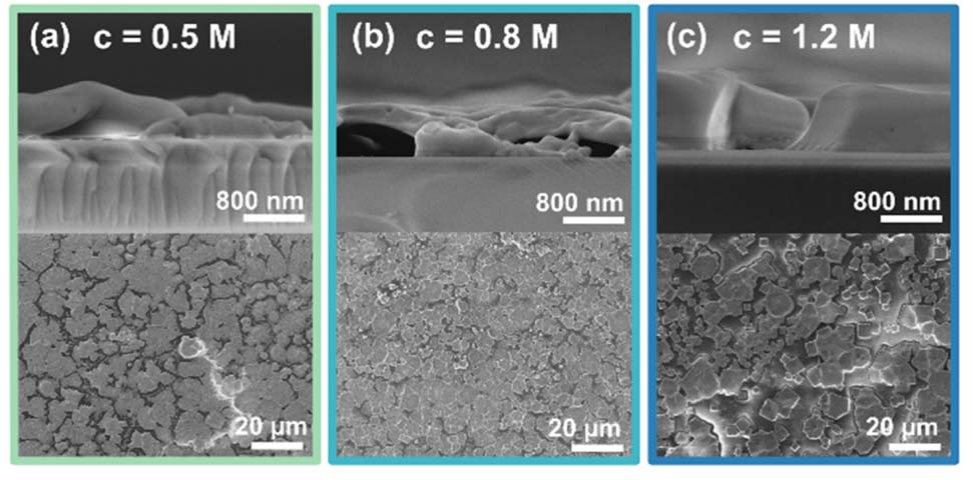
SEM images of the final 3CatPbBr_3_ films prepared from the solution concentration series, (a) 0.5 M, (b) 0.8 M, (c) 1.2 M. The top row presents the cross-section, while the bottom row presents the top view images. All three concentrations result in rough and incomplete films regarding coverage and morphology.

First, thin film characteristics are presented. The layers were prepared from a solution concentration series of 0.5 M, 0.8 M, and 1.2 M for preparation. Fig. 1 presents SEM images highlighting the morphology and thickness of the final FA_0.85_MA_0.10_Cs_0.05_PbBr_3_ (3CatPbBr_3_) thin films prepared from the precursor solution concentration series. All thin films prepared from the concentration series show a low coverage with non-connected grains. While grains formed from the 0.5 M solution exhibit an undefined shape, grains formed from the 0.8 M and especially from the 1.2 M solution are cubic-shaped. For all three samples, the film thickness is very inhomogeneous and rough. The film thickness decreases from 900–1000 nm for spin-coating the 1.2 M solution to 300–600 nm for the 0.5 M solution. The limited coverage causes the roughness of the films. However, with the varying film thickness, more material is deposited on the substrate using higher concentrated solutions.

Overall, SEM images demonstrate a low-quality morphology of the 3CatPbBr_3_ films of the concentration series. Setting an anti-solvent drop during spin-coating ensures a closed film with a high coverage and a wrinkled morphology (Fig. S2†). Although the morphology quality benefits from setting an anti-solvent drop, this parameter is not discussed in detail. Setting an anti-solvent drop is a specific technique limited to spin-coating. Herein, we will investigate and explore the solution chemistry and crystallisation kinetics independent of this deposition method. This study aims to rationalise the influence of the solution concentration. Thus, only an unperturbed formation process without setting an anti-solvent drop is discussed below. Setting an anti-solvent drop would result in higher-quality films by quenching the process but intervening with the intrinsic formation kinetics. Rationalising the unperturbed process is crucial to rationalise the entire formation of MHP. In a later step, it will help either optimise the anti-solvent drop, replace it with other methods, or develop coating processes where no classical anti-solvent drop is possible. Profound knowledge will be helpful for the inevitable transition to industrial-scale deposition methods like printing and coating techniques. However, the influence of setting an anti-solvent drop during spin-coating of the 1.2 M 3CatPbBr_3_ precursor solution is briefly presented in ESI Note 1 and Fig. S5.†

Fig. S3† summarises the optical and structural properties of the final 3CatPbBr_3_ films. As discussed by Tian *et al.*,^39^ the low-quality morphology of the films results in a feature-less, low absorption above the bandgap (Fig. S3(a)†). The absorption is positioned from 550 nm, and the PL emission is centred at 545 nm, while the sample prepared from the 1.2 M solution shows a minor shift to 550 nm. All samples show a PLQY range from 0.30–0.37%. The XRD pattern (Fig. S3(b)†) confirms the formation of the perovskite phase with reflections at 2*θ* = 14.8, 21.0, and 29.7, comparable to the positions of cubic FAPbBr_3_. An additional reflection at 2*θ* = 12.3 occurs. Due to missing further reflections with sufficient intensities, this secondary phase is not identifiable. Thus, revising the final 3CatPbBr_3_ film properties, the solution concentration mainly influences the thin film morphology. Overall, we hypothesise that the underlying reason for these differences is the crystallisation process, which determines the optoelectronic properties of the final samples, especially in high-quality films. Thus, in the following, we focus on the detailed rationalisation of the formation kinetics depending on the precursor solution concentration.

### Formation process and kinetics

**Fig. 2:**
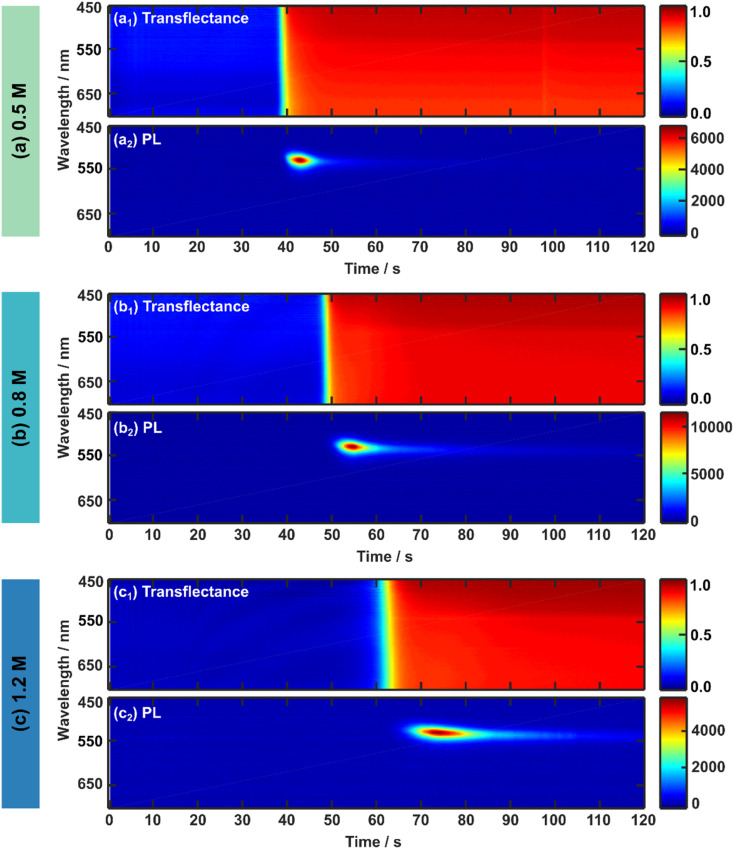
Optical *in situ* measurements of the 3CatPbBr_3_ solution concentration series during spin-coating. The 2D heat maps present the *in situ* data over 120 s of spin-coating for the (a) 0.5 M, (b) 0.8 M, and (c) 1.2 M solution. The upper panels (subscript 1) exhibit the UV-vis (transflectance) data, while the lower panels (subscript 2) exhibit *in situ* PL data. *In situ* PL is measured with an integration time of 500 ms. While transflectance measurements exhibit a high background signal hampering data analysis, *in situ* PL measurements display a clear signal evolution that is more straightforward for data analysis. Therefore, *in situ* UV-vis and PL measurements correlate, supporting and strengthening their results. For higher concentrated solutions, both *in situ* signals suggest a delayed crystallisation onset.

To investigate the intrinsic formation process and kinetics of the 3CatPbBr_3_ perovskite thin films by optical *in situ* metrology, spin-coating is done without an anti-solvent drop. While spin-coating parameters are kept constant for comparability, the spin time is increased from 40 s to 120 s compared to the recipe established by Kulbak *et al.*^35^ to follow the entire intrinsic formation process for all three concentrations. Fig. 2 presents the *in situ* UV-vis and PL measurements in 2D heatmaps while spin-coating for three 3CatPbBr_3_ solution concentrations, namely (a) 0.5 M, (b) 0.8 M, and (c) 1.2 M.

The respective upper panels (a_1_), (b_1_), and (c_1_) in Fig. 2 illustrate the evolution of the *in situ* UV-vis (transflectance signal) during 120 s of spin-coating for the 0.5 M, 0.8 M, and 1.2 M solution. Since absorption and scattering influence the *in situ* UV-vis signal based on the assembly of the reflectance probe (see Fig. S1†), this measurement mode is referred to as transflectance.^19^ Although a change in the transflectance signal evolves for all three concentrations during prolonged times of spin-coating, no straight absorption edge expected around 500–550 nm for pure bromide-based perovskite is clearly identified. A low change of red tones only minimally indicates the absorption edge since scattering dominates the mean transflectance signal (Fig. S4†) for all solution concentrations over the entire wavelength region. Therefore, only a minor part of the detected light undergoes a transmission process and explains the faintly visible absorption edge around 500–550 nm. Even though the amount of absorbed light increases slightly for a higher concentrated solution, its share is vanishingly small and does not give conclusive insight into the perovskite phase evolution. The implicating evolution of an absorption edge at 500–550 nm suggests the direct formation^33^ of the 3CatPbBr_3_, supported by a noticeable absorption edge formation by setting an anti-solvent drop (Fig. S5 and ESI Note 1†). The above-described low-quality morphology of the final 3CatPbBr_3_ films justifies the phenomenon of increased scattering accompanied by the low absorption upon crystallisation.

To rationalise the formation process of the 3CatPbBr_3_ system comprehensively, *in situ* PL measurements during spin-coating are presented in Fig. 2(a_2_), (b_2_), and (c_2_) for the 0.5 M, 0.8 M, and 1.2 M solution. *In situ* PL measurements give complementary insights into the perovskite formation process since the background of the PL signal is less influenced by scattering due to filter effects than *in situ* UV-vis measurements in the respective configuration. In general, light-induced phase segregation^40^ is avoided by choosing a purely bromide-based perovskite and, thus, can be neglected in this formation study. All three concentrations display a comparable evolution of the PL emission over time during spin-coating. Due to the direct perovskite crystallisation of pure bromides, a PL signal around 530 nm (corresponding to 2.35 eV, Fig. S6†) arises with a rapid increase in intensity, decreases fast, and vanishes over the spin-coating process. The higher the solution concentration, the longer the PL signal evolution progress takes. Changes in the *in situ* PL and UV-vis measurements arise roughly at the same time within one solution concentration. Interestingly, the signal change indicating the start of the formation process is delayed for higher concentrated precursor solutions. From these first observations, especially for *in situ* PL metrology, differences in the formation kinetics upon the solution concentration are hypothesised.

**Fig. 3:**
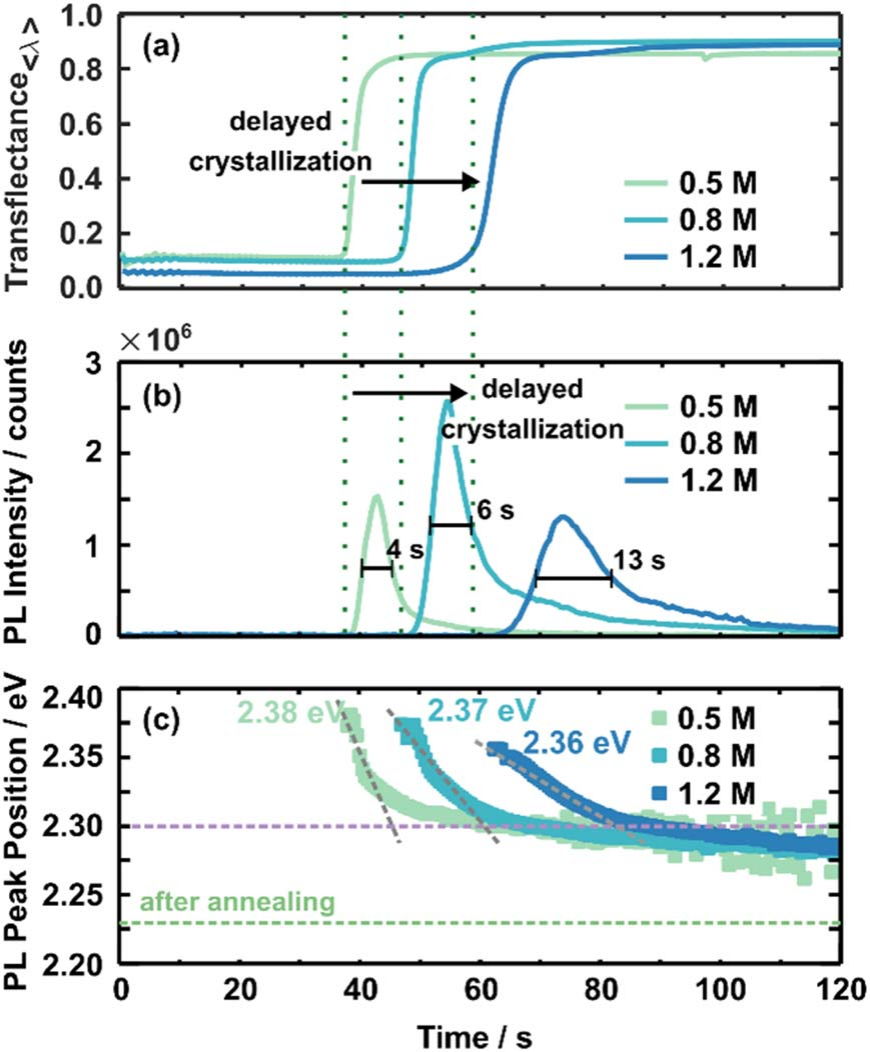
The averaged transflectance (a) and the PL intensity evolution (b) are compared for all three concentrations. A rise in transflectance and PL intensity indicates the onset of crystallisation, marked by green dashed lines for each concentration. Due to the correlative character of *in situ* UV-vis and PL measurements, the onset timing corresponds. Overall, higher concentrated solutions crystallise delayed. The evolution of the PL peak position is presented in (c). Values stated in eV give the initial PL peak position, and the grey dashed lines guides the eye to the slope of the shift in the PL peak position. The purple dashed line indicates the energy around this turnover point. The green dashed line indicates the PL peak position of the final 3CatPbBr_3_ films.

*In situ* PL metrology datasets are analysed and discussed in more detail (Fig. 3) to investigate the concentration-dependent formation kinetics. Fig. S6† presents the *in situ* PL measurements for the solution concentration series recalculated by Jacobians transformation^41^ in units of energy (eV). Peak analysis in the following uses this recalculated data.

As discussed above, scattering mainly influences *in situ* UV-vis measurements hiding a clear absorption edge. Thus, *in situ* UV-vis metrology mainly identifies the crystallisation onset by increased averaged transflectance (Fig. 3(a)). Increased averaged transflectance indicates a change in scattering due to the film solidification and, thus, translates to the crystallisation onset. For example, the crystallisation of the 0.5 M solution starts at ∼37 s within the spin-coating process, while increasing the concentration to 0.8 M and 1.2 M delays the crystallisation onset to ∼45 s and ∼60 s, respectively. At first glance, this time-dependent behaviour is contrary to an expected crystallisation from the LaMer model commonly utilised to describe perovskite crystallisation from solution.^37,42^ Within this model, super-saturation initialises nucleation and subsequent crystal growth. Higher concentrated solutions should reach the saturation limit faster upon solvent evaporation and, thus, crystallise earlier. These assumptions do not appear accurate for the presented 3CatPbBr_3_ system. In contrast, the crystallisation onset is delayed for higher concentrated solutions. Setting an anti-solvent drop during a relevant process window induces crystallisation (Fig. S5 and S7†). Here it must be noted that the dependency of formation kinetics on the solution concentration is influenced by the experimental design. Deliberately choosing a low boiling point solvent influences this behaviour and is a way to tune the formation process.^43^

Fig. 3(b) presents the evolution of the PL intensity at the leading peak position for the solution concentration series during spin-coating. A PL signal arising around 530 nm is attributed to the presence of the 3CatPbBr_3_ perovskite. Thus, the PL signal appearing at this wavelength translates to the formation of the desired perovskite. Furthermore, the rise in PL intensity for all three concentrations correlates well to the averaged transflectance (green dashed lines) increase at 38 s, 48 s, and 61 s for 0.5 M, 0.8 M, and 1.2 M solutions. Thus, PL measurements verify a delayed crystallisation onset for higher concentrated solutions, as discussed for *in situ* UV-vis measurements. Furthermore, slight differences in crystallisation time are attributable to measurements on different samples.

The fitted data confirms the considerable signal increase at the onset of crystallisation followed by an immediate decay before being observed in the 2D heatmaps (Fig. 3(b)). Based on this trend, we hypothesise the following nucleation and growth process: numerous tiny crystal nuclei or nucleation centres precipitate simultaneously during crystallisation induced by solvent evaporation. Similar to perovskite nanocrystals, such nucleation centres would result in the described substantial rise in PL intensity. Very rapid crystal growth of nucleation centres can explain the immediate decrease in PL intensity. During film formation, the film condenses more and more due to the growth of crystallites. The growth leads to defects and grain boundaries within the film and impacts the charge carrier diffusion and non-radiative recombination. These two effects occurring during film formation quench the PL intensity. A low PL intensity is associated with re-absorption by the already formed crystallites and a lower outcoupling caused by continued solidification^44^ during ongoing spin-coating. Optical effects like re-absorption and outcoupling are justified within the refractive index and morphology. A rough and low-quality morphology will increase diffusive reflection. Due to continuous growth, the wet film becomes more comparable to the thin film properties; thus, optical signatures change. Nevertheless, the measurement setup, *e.g.* the detector to sample distance, stays the same, and statements are valid during the crystallite growth based on relative changes.

Although the shape of the PL intensity follows the same trend for all concentrations, the maximum PL intensity and the time evolution differ with the concentration. The maximum PL intensity increases from 1.5 × 10^6^ counts for the 0.5 M solution to 2.5 × 10^6^ counts for the 0.8 M solution. This significant difference in maximum intensity is hypothesised to point to more nucleation centres forming during initial crystallisation. Interestingly, this trend does not continue for the 1.2 M solution with a reduced maximum PL intensity of 1.3 × 10^6^ counts. The reduced maximum intensity indicates the formation of fewer seed crystals or already larger nucleation centres. Seed crystals form more and more from colloidal structures and, thus, are limited in number. This phenomenon will be discussed in more detail below. Surprisingly, the time from the PL onset until reaching the PL maximum, indicated by the broader FWHM, extends with increasing solution concentrations, from 4 s for the 0.5 M, to 6 s for the 0.8 M, and 13 s for the 1.2 M solution. The retarded peak evolution for higher concentrated solutions suggests slower nucleation and crystal growth for higher concentrated solutions. The retarded nucleation and crystal growth accompany the delayed crystallisation onset. The decelerated formation kinetics explain the defined growth of cube-shaped crystallites for films prepared from higher concentrated solutions (Fig. 1).

Fig. 3(c) presents the evolution of the PL peak position for the three established 3CatPbBr_3_ solution concentrations. Irrespective of solution concentration, the evolution of the PL peak positions is consistent. It arises at higher energies, drops quickly to lower energies, and then stabilises after shifting slightly toward lower energies during ongoing spin-coating.

The initial high energy PL emission at 2.38 eV (0.5 M) to 2.36 eV (1.2 M) indicates larger nucleation centres forming from the higher concentrated solutions. Likely, nucleation centres demonstrate PL emission at higher energy, while the PL peak position shifts to lower energy during crystal growth. The PL progress supports that small nucleation centres form upon crystallisation and rapidly grow to larger crystallites. Overall, bigger nucleation centres tend to form for the higher concentrated solution. The initial PL peak position at 2.38 eV for the 0.5 M solution shifts to 2.36 eV for the 1.2 M one, correlating to crystallite sizes of 7.52 nm and 8.26 nm, extrapolating literature values of MAPbBr_3_ nanoparticles.^45^

The rapid shift in the PL peak position toward lower energies signals the crystal growth for all three concentrations until a turnover point is reached. After this, the PL peak position only shifts slightly to lower energies; thus, the crystal growth is slowed. Comparable to the nucleation onset discussed before, the timing of the turnover point is delayed for higher concentrated solutions, from 45 s for the 0.5 M solution within the spin-coating process to 85 s for the 1.2 M solution. Hence, the window for crystallite growth extends from 7 s for the 0.5 M solution to 24 s for the 1.2 M one. This increased growth window indicates a comparable slower crystallite growth within the fast growth regime directly after nucleation for a higher concentrated solution. The grey dashed lines with different slopes visualise this phenomenon in Fig. 3(c). The flatter the slope, the slower the crystallite growth. A slower and, thus, more defined crystallite growth explains the more defined cubic shape of the grains of the film spin-coated from the 1.2 M solution.

In addition, the turnover point time roughly corresponds to when the PL intensity starts vanishing for all three solution concentrations. This correlation in PL intensity and position emphasises that nucleation and crystal growth mainly occur in the first seconds after crystallisation. The same trend in decelerated nucleation and crystal growth process is derived from PL intensity and peak position though absolute timings vary due to different anchor points in both parameters. The fast shift in the PL emission to lower energies is also observed for pure, homogeneous crystallising MAPbBr_3_ (Fig. S7†). Thus, grain growth dominates this shift rather than inhomogeneities caused by the cation distribution.

Overall, re-absorption, acting as a filter effect, appears in perovskites and influences PL spectra. Due to their high absorption coefficients, the high-energy part of the PL spectrum is re-absorbed and, thus, cut off. The resulting sharp PL onset on the high-energy side becomes more important for ongoing formation since the filter effect increases with perovskite thickness. Additionally, a decay in PL intensity connects to increased re-absorption caused by continued solidification of the perovskite film.^46^ Even in the early stages, re-absorption affects the PL emission peak to a certain extent. Larger particles with a lower bandgap re-absorb the high-energy emission of smaller particles. Thus, the PL emission peak represents the maximum crystallite size at the early nucleation and crystal growth stages.

The PL peak position for all three concentrations is around 2.30 eV when the PL peak position stabilises, indicating a comparable crystal size at this stage of the formation process. However, at the end of the spin-coating process, the stabilised PL peak position lies at higher energy than the PL emission of the final 3CatPbBr_3_ films (2.23 eV, green dashed line). Slow, ongoing grain growth and residual polar solvent, causing a solvatochromic shift, are accountable for the offset. The annealing step finally removes residual solvents and completes the perovskite formation process. In addition, the wet-film thinning is also solution concentration-dependent. Overall, lower concentrated solutions result in thinner wet films (ESI Note 2 and Fig. S8 and S9†).

In Fig. S10,† we additionally present the *in situ* UV-vis data for the so-called “triple cation” (Cs_0.05_MA_0.17_FA_0.83_)Pb(Br_0.17_I_0.83_)_3_ perovskite. Also, for this perovskite composition, the lower concentrated solutions crystalise earlier. While the 0.8 M solution crystallises ∼60 s within the spin-coating process, the 1.2 M solution crystallises at ∼90 s. Exact timings and, thus, detailed kinetics differ between the “triple cation” iodide-based perovskite and the bromide-based archetypes. However, the same trend is demonstrated, and the kinetics discussed above for pure-bromide MHP can be transferred to other perovskite compositions. The delayed crystallisation for higher concentrated solutions is highly relevant for all perovskite compositions and optimising deposition processes for solar cells, LEDs and other optoelectronic applications. Therefore, it can be concluded that the kinetics analysed and the connected reasons in solution chemistry stay comparable for more complex perovskite compositions containing iodide. The formation kinetics of the bromide-based perovskite concentration series is deliberately investigated since those form directly from solution and optical complex to detect intermediate solvate phases, as expected for iodide-based compositions, can be excluded.

In summary, complementary *in situ* UV-vis and *in situ* PL measurements reveal a strong dependency of the 3CatPbBr_3_ formation kinetics on the precursor solution concentration. Higher concentrated solutions delay the onset of crystallisation and retarded nucleation and growth kinetics.

### Solution characteristics and chemistry

The solution concentration has a pronounced influence on perovskite formation kinetics. However, comprehensive rationalisation of the perovskite formation processes requires further insights into the respective solution chemistry. SAXS (Small Angle X-ray Scattering) measurements were performed to investigate the 3CatPbBr_3_ solution concentration series on a nanostructural level. The study by Flatken *et al*.^47^ demonstrates the applicability of SAXS measurements on perovskite solutions and presents the existence of structured colloids and their interaction in solution, resulting in pre-crystalline arrangements. The form factor gives insights into the shape and size of the nano-objects in the colloidal assembly itself. The shape, size and inter-particle interference, reflected in the structure factor, directly affect the scattering profile of the solution.

**Fig. 4:**
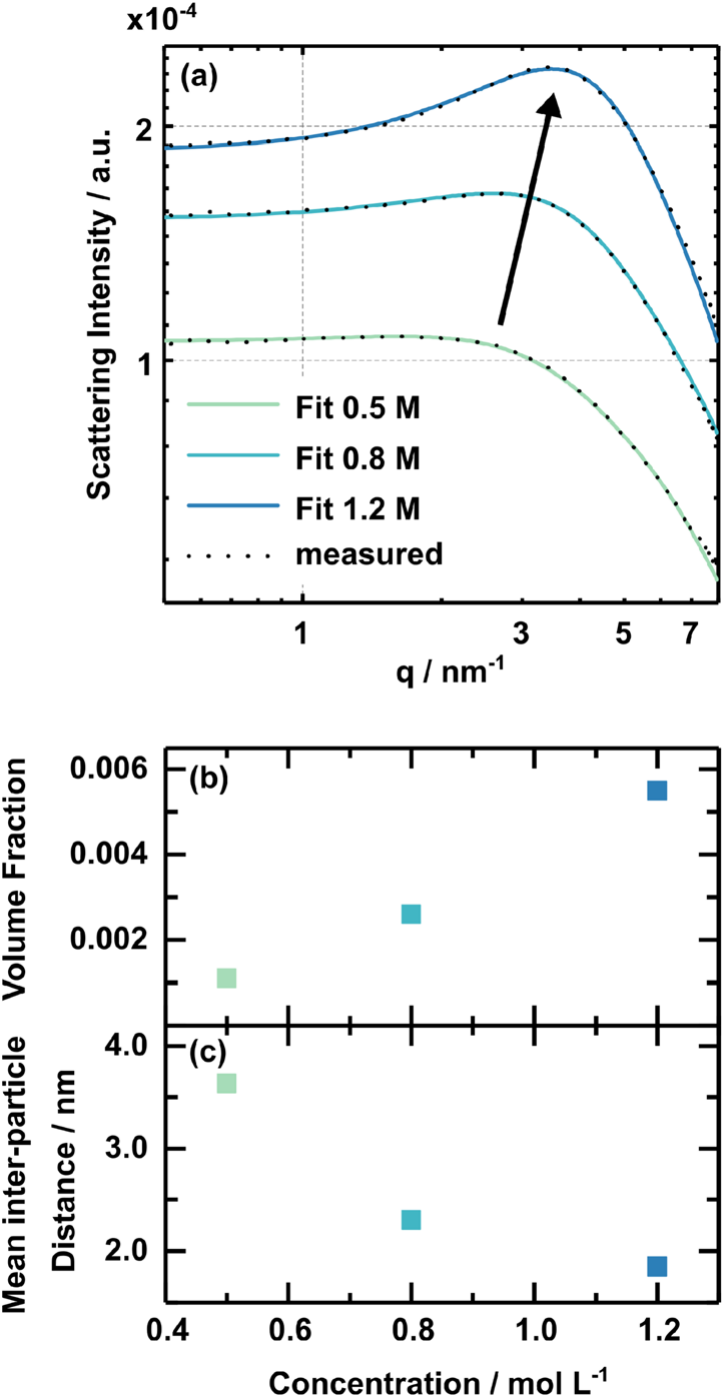
SAXS data on the 3CatPbBr_3_ solution concentration series. The scattering intensity is illustrated in (a) for 0.5 M, 0.8 M, and 1.2 M concentrated solutions with black dots representing the raw data and solid lines the fit. (b) Demonstrates the concentration dependency of the structural factor, namely the volume fraction and (c) the mean inter-particle distance. *R*-values for the fits are 0.0114 (0.5 M), 0.0033 (0.8 M) and 0.0038 (1.2 M).

Fig. 4(a) presents the scattering curves for the samples with 0.5 M, 0.8 M, and 1.2 M solution concentrations. The black dotted lines demonstrate the actual measured data, while the solid lines show the fit from the software SASfit©.^48^ A broad maximum evolves for higher concentrated solutions. This specific increase in scattering intensity demonstrates the domination of a structure factor and indicates an interaction, pre-organisation, of the observed particles in the solution. The stronger the maximum, the more particles are involved in forming these pre-organised clusters. The volume fraction derived, assuming a hard-sphere structure factor model, expresses the measure. Fig. 4(b) presents the concentration dependency of the volume fraction, increasing from 0.011 in the 0.5 M solution to 0.056 in the 1.2 M one. Thus, higher concentrated solutions possess a higher structural pre-organisation within the colloids.

Using the extended Bragg equation:^49^
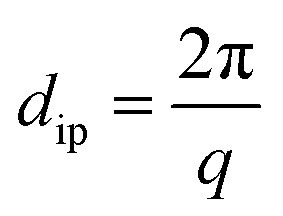
where *q* is the position of the peak maximum in *q*-space, the mean inter-particle distance (*d*_ip_) between the mass centres of the subunits within the colloids is described. Fig. 4(c) shows the concentration-dependent inter-particle distance. The mean inter-particle distance reduces from 3.63 nm in the 0.5 M solution to 1.85 nm in the 1.2 M one. Thus, the individual subunits, on average, come closer together and interact stronger within the higher concentrated 3CatPbBr_3_ solutions. The characteristics of the colloidal dispersion strongly influence the crystallisation process *via* repulsive interaction between colloids and, by that, the colloidal stability. Non-classical nucleation and growth mechanisms can explain these phenomena.^50^

**Fig. 5:**
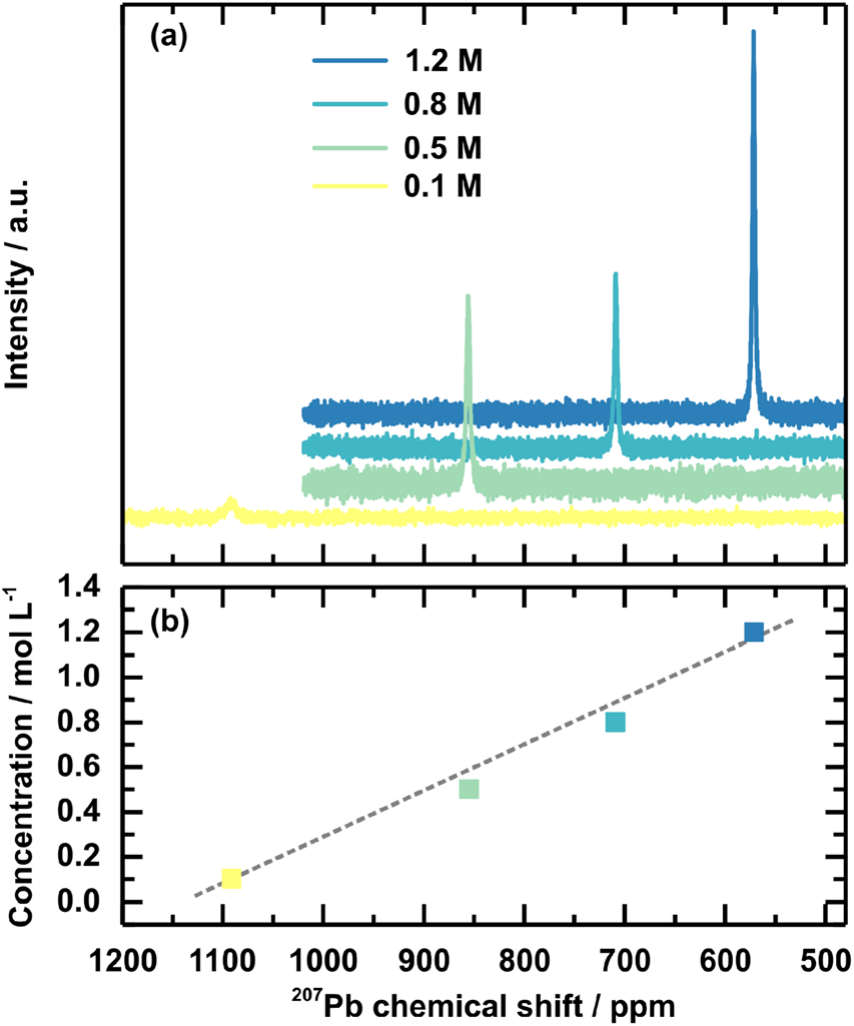
^207^Pb NMR spectra (a) of the 3CatPbBr_3_ solution concertation series and an additional diluted 0.1 M solution. (b) Visualises the dependency of the ^207^Pb chemical shift on the concentration. The grey dashed line gives a guide for the eye of the linear dependency.

In addition, ^207^Pb NMR measurements on the solution concentration series were performed to investigate the chemical environment of the Pb^2+^ ion within the respective solutions. An upfield shift to lower ppm values is observed for increased solution concentration (Fig. 5). In comparison, the peak for the 0.1 M solution is at 1091 ppm, and the peak for the 1.2 M solution shifts to 571 ppm. The peak shifts linearly to the solution concentration (Fig. 5(b)). Interestingly, the chemical shift of the 1.2 M solution shifts close to the peak position for solid-state ^207^Pb NMR of FAPbBr_3_ at 515 ppm.^51^ FAPbBr_3_ is chosen as the reference value since FA^+^ is 85% of the cations in the discussed example. Overall, these chemical shifts confirm a shifted chemical equilibrium. The upfield shift upon the increased solution concentration is connected to an increased electron density around the lead. This effect firstly can indicate an agglomeration of the individual Pb^2+^ into a network, which the previously discussed SAXS measurement verified by the described higher pre-coordinated structures. Since the chemical shift of the 1.2 M concentrated solution and the solid-state FAPbBr_3_ are comparable, their chemical environments are also comparable in terms of coordination sphere, coordination number, and ligands. This comparison supports the interpretation of a higher pre-ordering of clusters in higher concentrated precursor solutions, comparable to the environment in the final perovskite, with more electron-donating Br^−^ as ligands coordinating the lead.

In addition, solution-based UV-vis measurements indicate a chemical shift to higher bromide-coordinated lead-halide-solvent complexes or the formation of poly-complexes (Fig. S11 and ESI Note 3†). Thus, a third solution-based measurement technique also confirms a shift in the chemical equilibrium.

## Conclusion

Changing the precursor solution concentration of MHP precursor solution not only decreases the thin film thickness but also results in considerable changes in the formation mechanism and kinetics. Lower concentrated solutions exhibit accelerated formation kinetics, namely earlier nucleation and faster crystallite growth, translating into a narrow process window for lower concentrations. Therefore, controlling the film formation process for lower concentrated solutions gets more complex, potentially leading to lower quality thin films and a decreased device performance.

We showed that the solution chemistry also depends on the precursor concentration, the chemical equilibrium shifts with the increase of the precursor solution concentration. Higher concentrated solutions possess a higher structural pre-organisation in colloids interacting more strongly with each other. Thus, the concentration defines the precursor solution fundamentally, such as the chemical interaction and pre-organisation of precursor salts and solvents. Overall, the solution chemistry predetermines the formation process and kinetics. Thus, the precursor solution unveils excellent potential to optimise the perovskite thin film deposition from solution-based techniques, *e.g.*, by new precursor and solvent combinations. This gets especially important for elaborate precursor compositions and possible intermediate phases forming for iodide-based perovskites. Hence, a detailed look at the precursors over the solution chemistry, the individual formation pathways and kinetics to the thin film properties are necessary to rationalise the formation processes of MHPs fully. Furthermore, the precursor solution concentration should be maintained while optimising the thin film thickness, not to alternate the underlying solution chemistry and formation kinetics.

The direct insights into the detailed formation kinetics depending on the precursor solution concentration demonstrate the significance of *in situ* metrology. Interestingly, small, partly unconscious changes in the preparation process enormously impact the overall formation of MHPs and their thin film quality. Thus, *in situ* metrology allows for uncovering crucial preparation parameters, leading to detailed, standardised preparation routines.

## Conflicts of interest

There is no conflicts of interest to declare.

## Supplementary Material

RA-012-D2RA06314J-s001
