# Supplementary material for: Role of solution concentration in formation kinetics of bromide perovskite thin films during spin-coating monitored by optical in situ metrology
Source: RSC Adv. 2022 Nov 15;12(50):32765–74. doi: 10.1039/d2ra06314j (PMC9664315; doi:10.1039/d2ra06314j)
Supplement: RA-012-D2RA06314J-s001 [file RA-012-D2RA06314J-s001.pdf]

Electronic Supplementary Material (ESI) for RSC Advances.
This journal is © The Royal Society of Chemistry 2022

Supporting Information

Role of solution concentration of formation kinetics of bromide perovskite thin films during spin-coating monitored by in-situ optical metrology

C. Rehermann[1], V. Schröder[2], M. Flatken[3], F. Ünlü[4], O. Shargaieva[1], A. Hoell[2], A. Merdasa[5], F. Mathies[1], S. Mathur[4] and E. L. Unger[1,6,7]

[1] Department of Solution-Processed Materials and Devices, HySPRINT Innovation Lab, Helmholtz-Zentrum Berlin für Materialen und Energie GmbH, Kekuléstraße 5, 12489 Berlin, Germany.

[2] Helmholtz Zentrum für Materialien und Energie GmbH, Hahn-Meitner-Platz 1, 14109 Berlin, Germany.

[3] Department Novel Materials and Interfaces for Photovoltaic Solar Cells, HySPRINT Innovation Lab, Helmholtz-Zentrum Berlin für Materialen und Energie GmbH, Kekuléstraße 5, 12489 Berlin, Germany.

[4] Inorganic and Materials Chemistry, University of Cologne, Greinstr. 6, 50939 Cologne, Germany.

[5] Department of Clinical Sciences Lund, Lund University, Sölvegatan 17, Lund, Sweden.

[6] Hybrid Materials: Formation and Scaling, IRIS Adlershof, Am Großen Windkanal 2, Humboldt Universität zu Berlin, 12489 Berlin, Germany.

[7] Chemical Physics and Nano Lund, Lund University, Lund, Sweden.

Experimental:

Substrates (microscope slides) were cleaned consecutively for 10 min with an alkaline detergent, acetone, and isopropanol in an ultrasonic bath. Subsequently, the substrates were dried via an N_2 flow and, directly before layer deposition, chemically cleaned by O_3-plasma for 20 min.

All following preparation steps were performed under inert conditions in N_2-filled gloveboxes. For the solution concentration-dependent formation study, a mixed cation, bromide-based perovskite was investigated and introduced by Kulbak *et al.*[1], containing MA^+, FA^+, and Cs^+ as A^+ site cations. The exact composition is given in Table S1. Instead of $FA_{0.85}MA_{0.10}Cs_{0.05}PbBr_3$, this perovskite is stated as $3CatPbBr_3$ for easier readability.

Table S1: Details of solutions prepared for the concentration-dependent formation study, with precursors, their ratio, concentration, and solvents utilized.

Solution	Precursor - ratio	Concentration / mol L^{-1}	Solvent
$PbBr_2$	-	1.2	7:3 DMF:DMSO
$MAPbBr_3$	$MABr:PbBr_2$ - 1:1	1.2	7:3 DMF:DMSO
$FAPbBr_3$	$FABr:PbBr_2$ - 1:1	1.2	7:3 DMF:DMSO
$3CatPbBr_3$	$FABr:MABr:CsBr:PbBr_2$ - 0.85:0.10:0.05:1.1	1.2	7:3 DMF:DMSO
$3CatPbBr_3$	$FABr:MABr:CsBr:PbBr_2$ - 0.85:0.10:0.05:1.1	0.8	7:3 DMF:DMSO
$3CatPbBr_3$	$FABr:MABr:CsBr:PbBr_2$ - 0.85:0.10:0.05:1.1	0.5	7:3 DMF:DMSO
$3CatPbBr_3$	$FABr:MABr:CsBr:PbBr_2$ - 0.85:0.10:0.05:1.1	0.1	7:3 DMF:DMSO

For the $3CatPbBr_3$ solution, first, a $PbBr_2$ stock solution was prepared. $MAPbBr_3$ and $FAPbBr_3$ solutions were realized by mixing corresponding amounts of the $PbBr_2$ solution with the respective ABr salt. Since CsBr has comparatively low solubility, the CsBr was directly mixed with corresponding amounts of $FAPbBr_3$, $MAPbBr_3$, and additional $PbBr_2$ solutions to obtain the final 1.2 M $3CatPbBr_3$ solution. According to Kulbak *et al.*[1] 10 % excess of $PbBr_2$ is added to the solution. The 1.2 M $3CatPbBr_3$ solution was diluted for the concentration series with corresponding amounts of 7:3 DMF:DMSO to obtain concentrations of 0.8 M, 0.5 M, and 0.1 M.

The solubility limit of CsBr is ~0.25 M in pure DMSO[2]. Thus, a comparative approach to Saliba *et. al.*[3] using a CsBr stock solution for the mixed cation solution does not work. As described above, directly dissolving CsBr in the mixture of $MAPbBr_3$ and $FAPbBr_3$ solutions demonstrates a work-around to this solubility issue. Thus, a solvent-free, mechanochemical introduction of the CsBr into the $3CatPbBr_3$ as presented by Saliba *et al.*[4] as the first step with the further dissolution of the resulting $3CatPbBr_3$ perovskite powder is not required. Therefore, working with Cs-containing pure bromide perovskites is possible without the special equipment for the solid-state synthesis (mills). In addition, this solution-based work-around was already introduced by Kulbak *et. al.*[1], illustrates the different mechanisms of solution chemistry for

pure CsBr and CsBr-containing perovskite solution. CsBr is presumably an additional Br^- source in the perovskite precursor solution. Higher coordinated lead-bromide complexes or clusters incorporate the additional Br^- and, hence, increase the solubility of CsBr in perovskite precursor solutions compared to pure CsBr only interacting with polar DMSO.

To investigate the influence of the solution concentration on the formation process of $3CatPbBr_3$, the spin-coating and annealing parameters were kept the same within the concentration series, as stated in Table S2:

Table S2: Details of perovskite thin-film preparation applied to study the concentration dependency of the film formation of $3CatPbBr_3$, with the amount of solution, spin-coating, and annealing parameters.

Amount of solution / µL	Spin speed / rpm	Acceleration / rpm s^{-1}	Spin time / s	Annealing Temp. / °C	Annealing time / min
60	1000 | 4000	1000 | 4000	10 | 30	100	30

The spin-coating process was performed in two steps, a slow one at 1000 rpm and a subsequent fast one at 4000 rpm. Only for the 1.2 M solution, an anti-solvent drop of 150 µL toluene was set after 17 s within the spin-coating process. Thus, for this concentration, the influence of an anti-solvent drop is described below in this Supplementary Information (SI Note 1). The films were thermally annealed directly after finishing the spin-coating.

To rationalize the formation kinetics of the $3CatPbBr_3$ perovskite, the spin-coating was monitored by in-situ UV-vis and in-situ PL measurements. Figure S1 is a schematic of the home-built optical in-situ set up inside the glove box. For detailed information on the measurement technique, refer to Merdasa *et al.*[5].

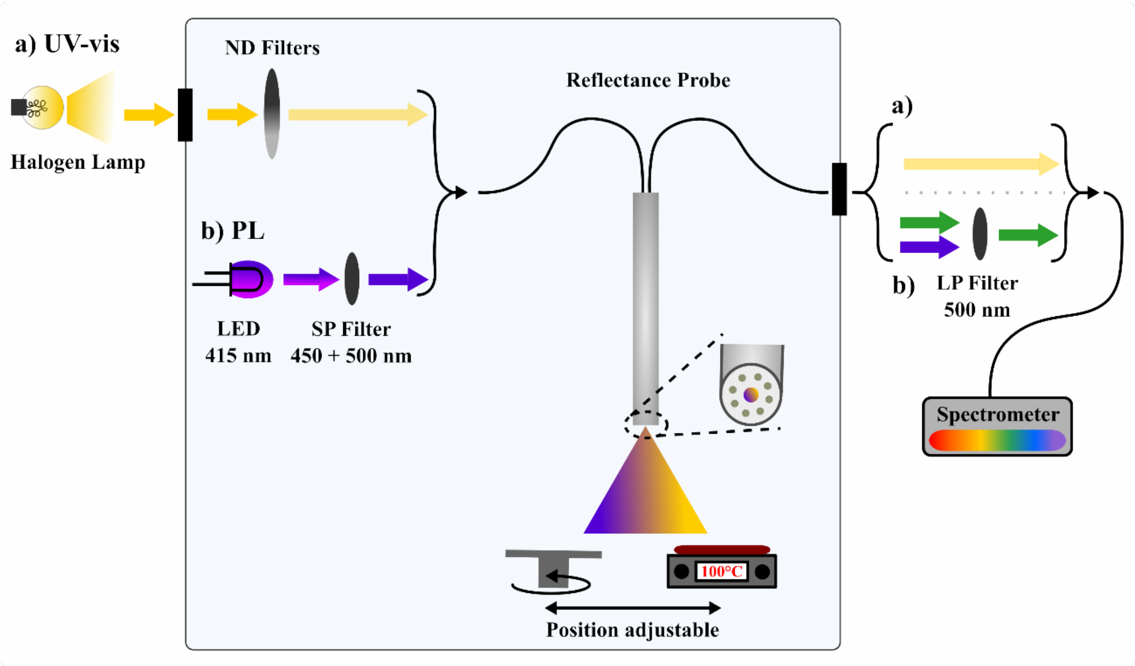

Figure S1: Schematic of the in-situ spectroscopy setup. The centerpiece is the reflectance probe adjustable above the spin-coater or the hot plate to monitor the respective process. (a) represents the light pass for the in-situ UV-vis measurements, using a halogen lamp as the excitation source with ND filters to adjust the light intensity, (b) indicates the light pass for PL measurements. Short and long-pass filters are utilized to cut off disruptive parts of the excitation from an LED. Components illustrated within the light blue box are assembled inside the glovebox and connected via optical feedthroughs to the components outside the glovebox.

Optical in-situ measurements - Substrates with an Ag mirror were utilized for in-situ UV-vis measurements. Depending on the orientation of the Ag mirror, either the absorption edge evolution or the thinning behavior was investigated. The halogen lamp was turned on 30 min before starting the measurements to ensure a stable emission spectrum. Individual spectra were collected with an integration time of 500 ms. Since no significant influences in terms of phase segregation are expected for pure bromide-based perovskites, in-situ PL analysis is straightforward in this case. However, integration times for individual spectra needed to be adjusted for every measurement, in consequence of strongly different PLQY, especially when applying an anti-solvent drop, to avoid oversaturation of the spectrometer. During spin-coating, 100, 200, and 500 ms integration times were applied. No Ag mirror on the substrates was utilized due to possible oversaturation.

Scanning Electron Microscopy (SEM) - SEM images were taken on a Hitachi S4100 with a cold field emitter and an acceleration voltage of 5 kV.

X-ray diffraction (XRD) - XRD measurements were carried out in air on a Bruker Advanced D8 in Bragg-Brentano geometry with a CuKα X-ray source ($\lambda = 1.5406$ Å).

UV-vis Spectroscopy (UV-vis) - UV-vis measurements on perovskite thin films were realized with a Lambda1050 system from PerkinElmer within an Ulbricht sphere. In contrast, UV-vis measurements on the precursor solutions were carried out on a Cary 5000 UV-Vis-NIR from Agilent utilizing demountable cuvettes from Hellma Analytics (type 106-QS) with quartz glass windows permeable from 200 – 2500 nm and a path length of 10 (± 3.0) µm.

Photoluminescence Quantum Yield (PLQY) - PLQY measurements were carried out in a home-built integrating sphere with 415 nm excitation (Thorlabs M415F3) at $30 mW/cm^2$, establishing 1 sun equivalent illumination conditions. The measurements consist of two consecutive measurements. First, the excitation without a sample is measured (I_0), and second, the remainder of the excitation (I_r) and the emission (I_{PL}), now with the sample mounted, is measured with a fiber-coupled spectrometer (OceanOptics, QEPro). By comparing absorbed vs. emitted photons, the PLQY is calculated as:

$$PLQY = \frac{I_{PL}}{(I_0 - I_r)}$$

The system is calibrated before measurements with a calibration lamp (Avantes AvaLight-CAL(-Mini)).

Small Angle X-ray Scattering (SAXS) - SAXS data were measured at the four crystal monochromator beamline in the PTB (Physikalisch-Technische Bundesanstalt) laboratory at BESSYII.[6] The sample to detector distance is variable without breaking the vacuum due to an adjustable about 3 m long support structure with a long edge-welded bellow.[7] A Dectris 1M PILATUS2 in-vacuum hybrid-pixel detector collects the two-dimensional scattering images. The measurements were carried out at energies of 10 keV and 8 keV, and at two distances of 0.8 m and 3.7 m, respectively. Thus, a q-range from 0.02 - 8.5 nm^{-1} was covered. The sample exposure time was set to 600 s with two repetitions for the short and three for the long distance. 0.1 mm thin, rectangular borosilicate cuvettes (CM Scientific, UK) were utilized due to the low transmittance of the lead-containing perovskite precursor solutions.
Data processing: The BerSAS software[8] was utilized for data reduction in radial averaging into the 1D pattern. All SAXS curves of the investigated samples were fitted with the program SASfit[9]. In order to get a general idea of the order of magnitude, a structure model that includes a spherical form factor and a hard-sphere structure factor was chosen to fit all sample scattering curves. The herein investigated particles were considered hard spheres. These particles are assumed incompressible, resulting in fixed radii for each particle and an infinite repulsive force at a certain interparticular separation. The hard spheres model neglects attractive forces but describes a various number of colloids in organic solvents fairly well.[10–12] We used the monodisperse Percus-Yevick approximation for hard spheres to fit the interaction of the analyzed colloidal particles.[13,14]

Nuclear Magnetic Resonance (NMR) - Nuclear magnetic resonance (NMR) spectra were measured with a Bruker AVANCE III 500 spectrometer. The chemical shifts were referenced externally to a solution of 1.0 M $Pb(NO_3)_2$ in D_2O ($\delta = -2986.3$ ppm) and are reported relative to $Pb(CH_3)_4$ in toluene ($\delta = 0$ ppm). Solutions for the NMR measurements were prepared freshly in a nitrogen-filled glovebox, filled into a glass NMR tube, and measured at 25 °C.

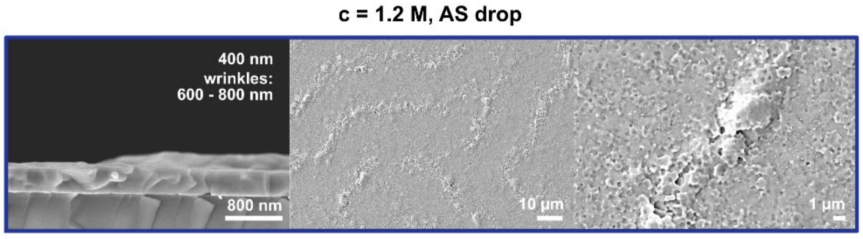

Figure S2: SEM images of a $3CatPbBr_3$ film prepared from a 1.2 M solution setting an anti-solvent drop. The left image presents the cross-section, while the middle and left present top view images at two different magnifications. For setting an anti-solvent drop during spin-coating, closed films with a high coverage form, however, those films show a wrinkled morphology.

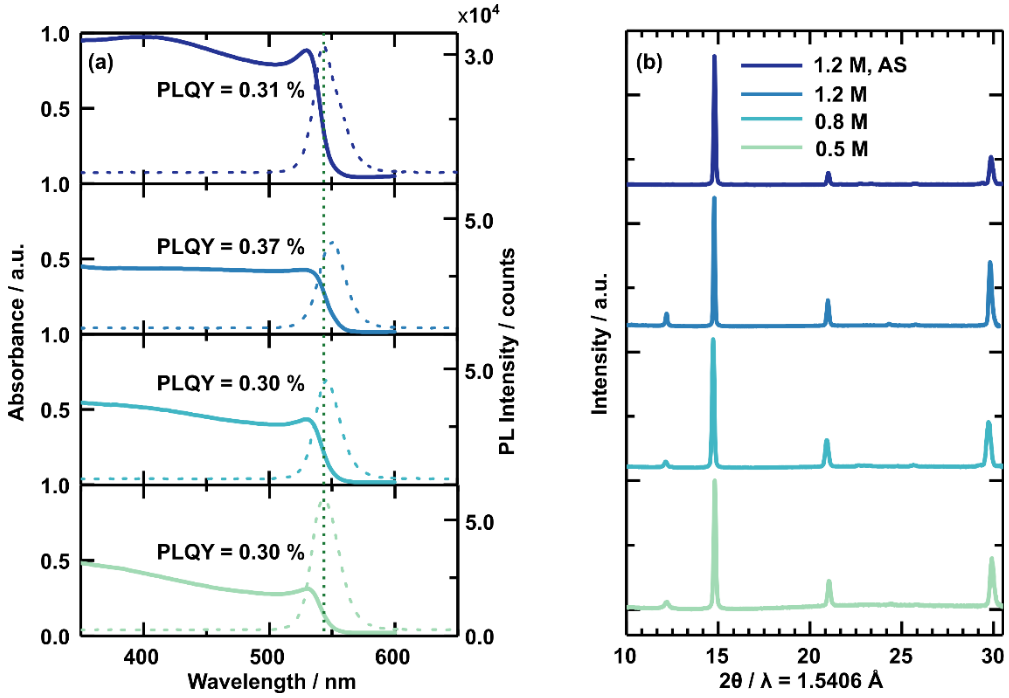

Figure S3: Comparison of final $3CatPbBr_3$ film properties of the established solution concentration series. UV-vis and PL spectra with the PLQY (a) and the XRD pattern (b) are presented.

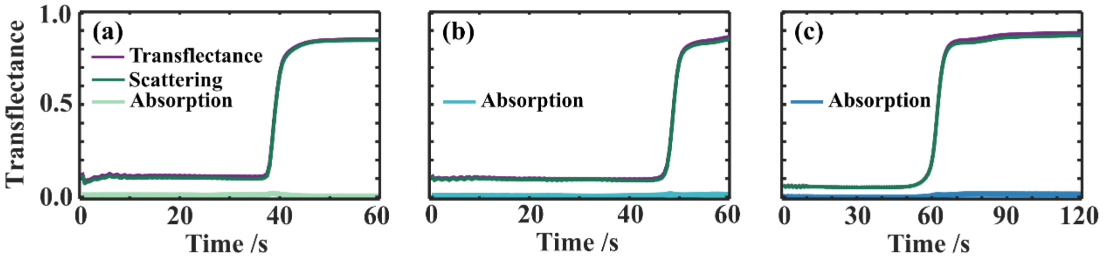

Figure S4: UV-vis measurements on the concentration series of $3CatPbBr_3$ solutions during spin-coating. The averaged transflectance from 450 – 800 nm (purple) over 60 s or 120 s of spin-coating is presented for the (a) 0.5 M, (b) 0.8 M, and (c) 1.2 M solution with their fitted scattering (green) and absorption shares.

SI Note 1:

Figure S5 summarizes the optical in-situ measurements, namely UV-vis, PL, and interference-sensitive measurements during spin-coating for the 1.2 M concentrated solution with setting an anti-solvent drop 17 sec within the spin-coating process. Fundamental differences appear in the signal evolution for all three measurements compared to the intrinsic formation process without setting an anti-solvent. The in-situ UV-vis and the in-situ PL heat map (Figure S5 (a) and (b)) indicate an induced crystallization by dropping the anti-solvent (green dashed line) based on the rise of the respective signal. A more detailed look at the mean transflectance and the PL intensity (Figure S5 (d)) confirms the induced crystallization.

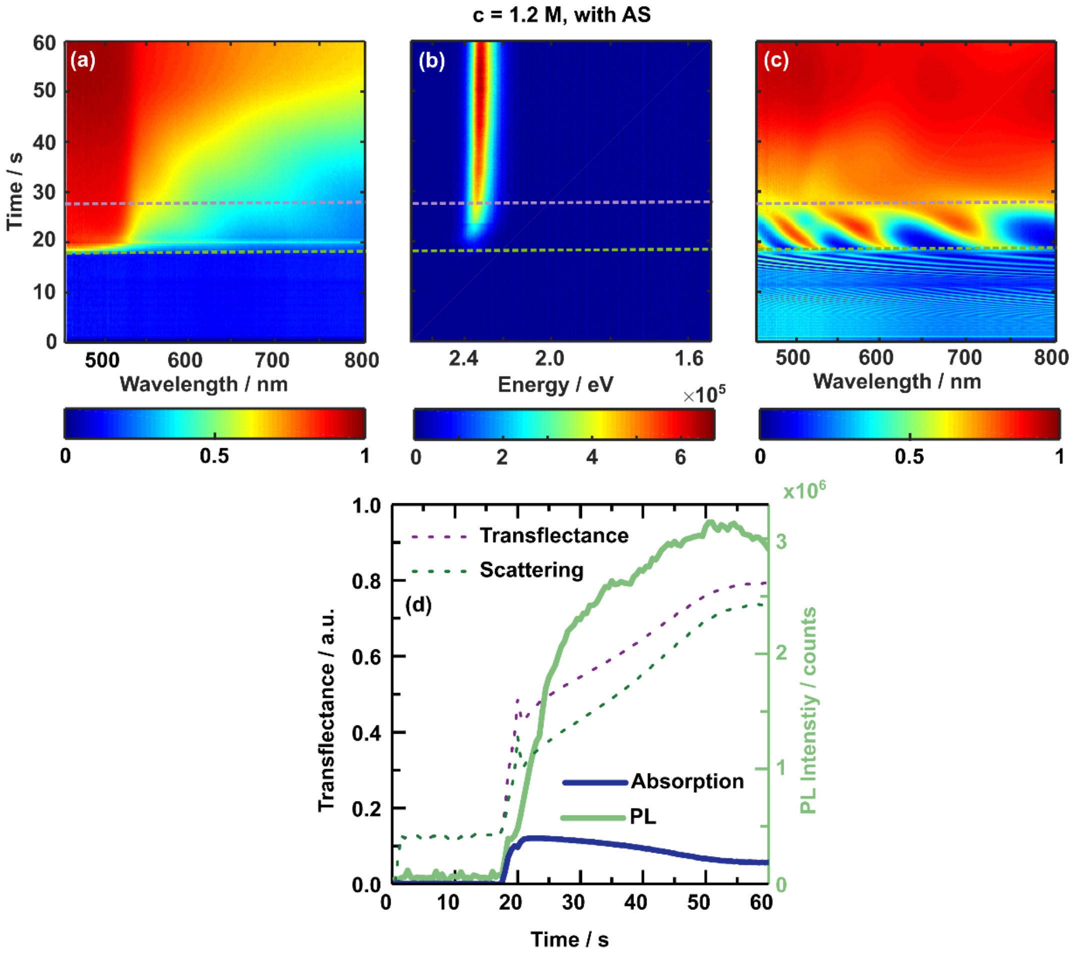

Figure S5: 2D heat maps of (a) in-situ UV-vis, (b) in-situ PL measurements, and (c) interference-sensitive in-situ UV-vis measurements during spin-coating of the 1.2 M 3CatPbBr$_3$ solution with dropping the anti-solvent 17 s within the process (light green dashed line). The purple dashed line indicates when no thinning of the wet film is detected anymore. The shares of scattering and absorption of the averaged transflectance, and the PL intensity evolution over the spin-coating process are shown in (d).

A clear absorption edge evolves after setting the anti-solvent. The absorption share of the overall transflectance increases in this case (Figure S5 (d)) and allows deriving the bandgap evolution. The initially low background after dropping the anti-solvent indicates the formation of a very smooth surface and a closed wet film, preventing scattering. However, around 27 s (purple dashed line) the background rises due to increased scattering and lowers the absorption

share. The enhanced solidification of the film and the growth of a wrinkled structure (Figure S2) justify increased scattering.

In-situ PL measurements do not present the short, pointy shape of the PL evolution discussed for not dropping an anti-solvent. The PL signal persists after dropping the anti-solvent over the complete spin-coating process. The integration time is reduced to 100 ms due to a much higher PL intensity. Figure S5 (d) presents the PL intensity recalculated to 500 ms integration time to compare the PL intensity at the peak center. This brief review of the PL evolution indicates a modification in the detailed formation process and kinetics by applying an anti-solvent drop.

The PL intensity evolution displays a maximum PL intensity of 3.1×10^6 counts, while the maximum counts are limited to 1.3×10^6 without an anti-solvent drop. The formation of smaller or more seed crystals and a greater amount of residual solvent in the film can explain this significant increase in the PL intensity. The PL intensity reveals a steep increase over ~10 s, comparable to the steep increase for films without an anti-solvent drop, until 27 s within the process. After that, however, the PL intensity rises lower until 50 s of spin-coating before gently decreasing. This long progress of the PL signal indicates slowed-down nucleation and crystal growth.

Figure S5 (c) demonstrates the thinning behavior for the 1.2 M solution by applying an anti-solvent. The interference pattern equals one of the 1.2 M solution without setting an anti-solvent in the beginning. However, dropping the anti-solvent causes a second abrupt modification in the interference pattern (green dashed line). Although the anti-solvent induces crystallization, an interference pattern with fewer fringes is detected. Only 27 s within the spin-coating process, scattering arising from ongoing crystallization starts to dominate the signal. Thus, setting an anti-solvent drop seems to reduce the wet film thickness.

Setting an anti-solvent drop induces the perovskite crystallization at the wet film surface and formation kinetics appear to slow down. The slowed-down crystallization process might induce the formation of a wrinkled morphology.

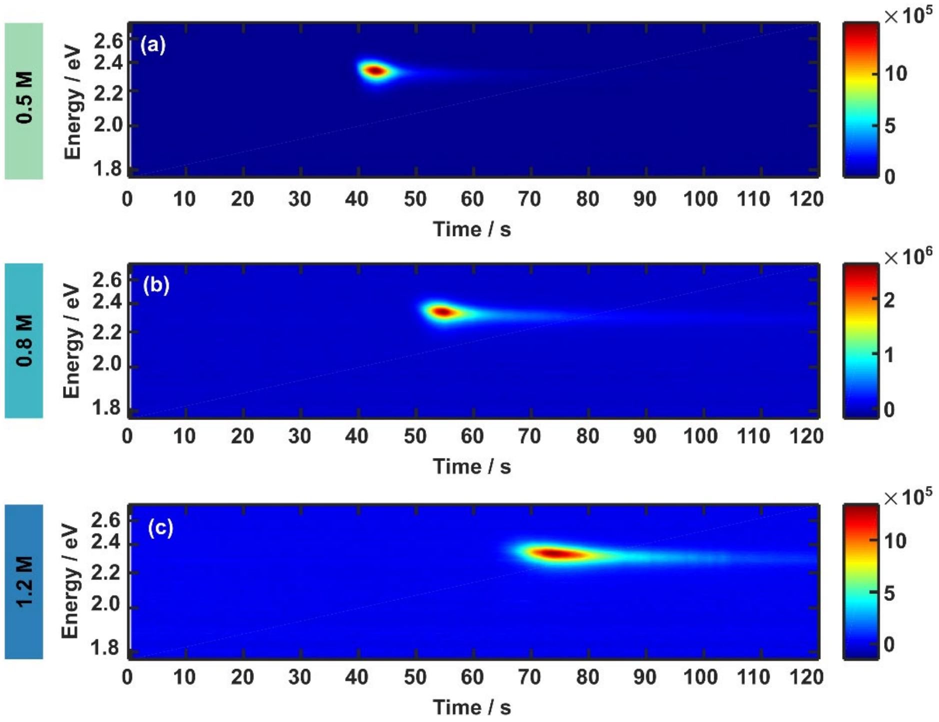

Figure S6: In-situ PL measurements of the 3CatPbBr$_3$ solution concentration series during spin-coating. The 2D heat maps present the in-situ data over 120 s of spin-coating for the (a) 0.5 M, (b) 0.8 M, and (c) 1.2 M solution. In-situ PL data from Figure 2 are recalculated in the energy scale using Jacobian conversion[15] for further fitting and data analysis. The energy scale covers the range from 1.55 - 2.75 eV, equivalent to 450 - 800 nm wavelength, as seen in Figure 2.

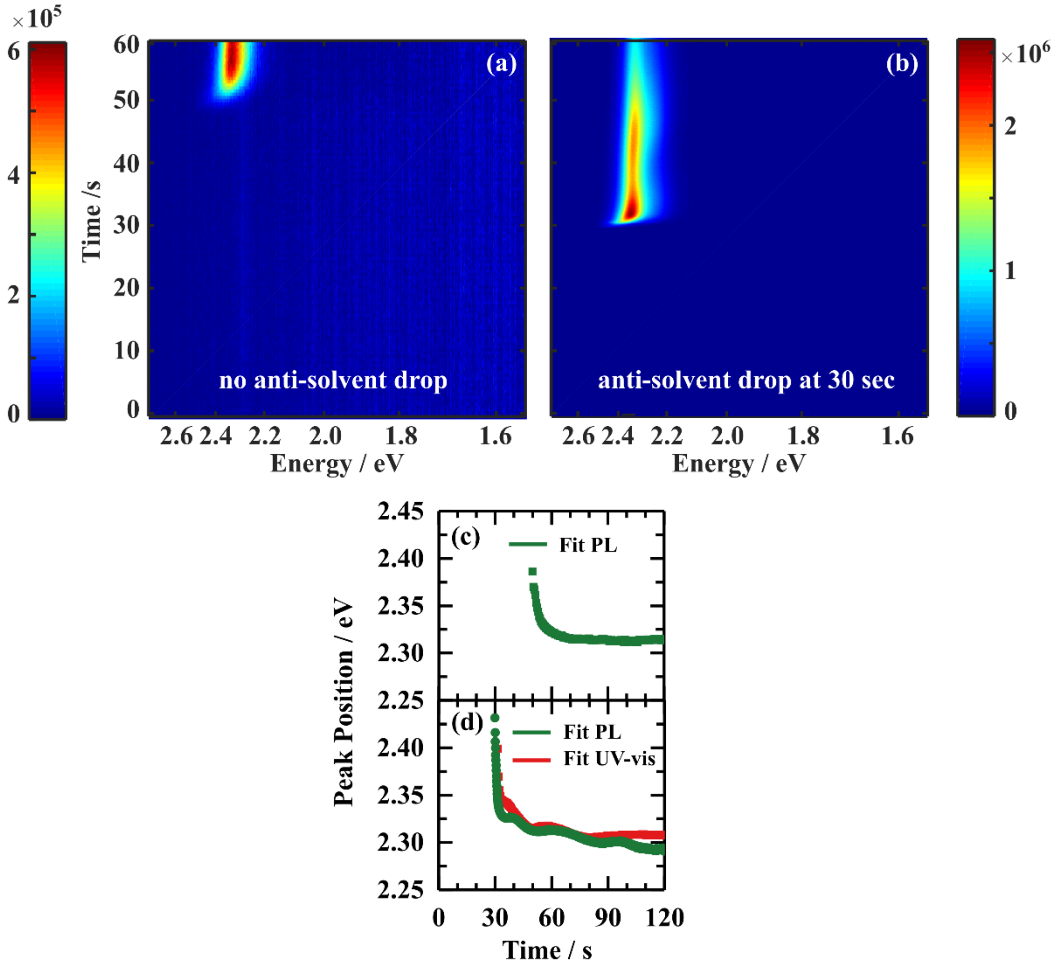

Figure S7: 2D heat maps of in-situ PL measurements presenting the formation process of $MAPbBr_3$ without (a) and (b) with dropping an anti-solvent at 30 sec tracked over 60 s of spin-coating. The PL peak position (c) for the $MAPbBr_3$ formation without an anti-solvent drop is shown in (c), while (d) represents the same parameter for setting an anti-solvent.

SI Note 2:

Additional in-situ UV-vis measurements sensitive to interference patterns provide insights into wet-film thinning for 0.1 M, 0.5 M, 0.8 M, and 1.2 M concentrated solutions. Figure S8 presents the interference pattern evolving during the spin-coating process. Independently from the solution concentration, a high number of fringes causes a delicate interference pattern during the initial period of spin-coating. The acceleration from 1000 to 4000 rpm at 10 s within the process evokes an abrupt modification in the interference pattern (dashed pink lines). Spinning at a faster speed reduces the number of fringes, and the interference pattern starts to deviate strongly as a function of solution concentration. The higher the concentration of the solutions, the slower the number of fringes reduces. As soon as crystallization starts, the interference pattern is not resolved anymore since scattering dominates the detected signal. Crystallization onsets from interference-sensitive in-situ UV-vis measurements demonstrate consistency in timing with the optical in-situ measurements discussed in detail in the main manuscript. For the 0.1 M solution, scattering dominates after 25 s within the spin-coating process, indicating the accelerated crystallization onset for this lower concentrated solution.

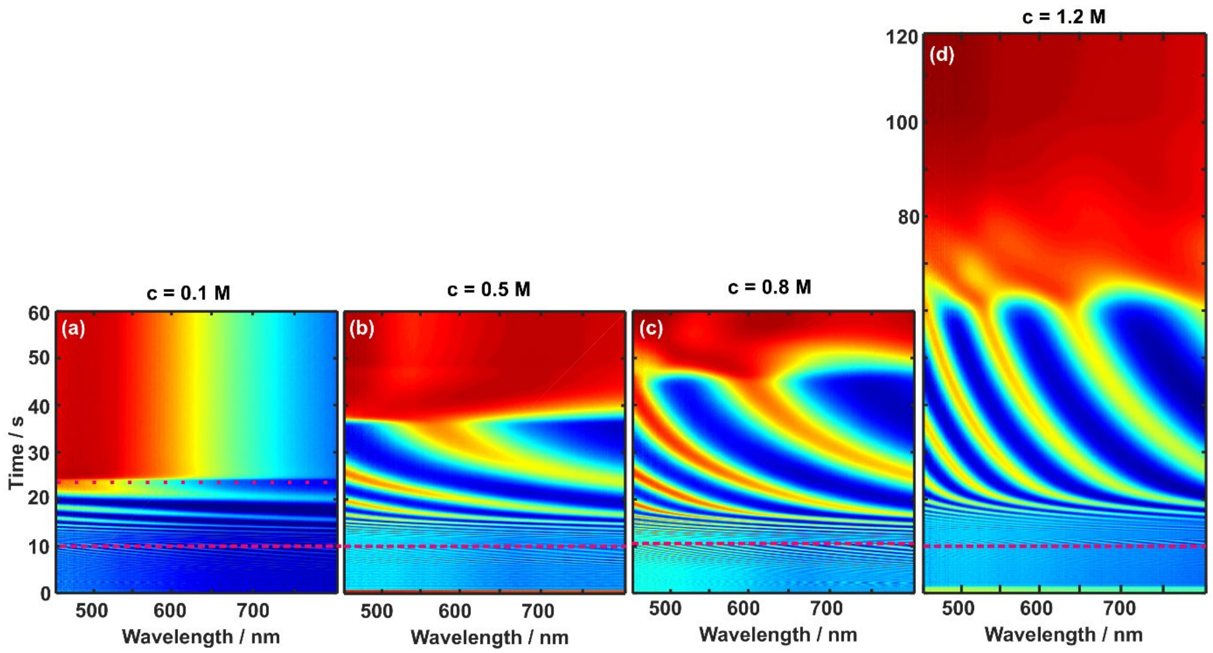

Figure S8: 2D heat maps of in-situ UV-vis measurements during 60 s or 120 s of spin-coating. Since the solutions are directly spin-coated on a silver mirror, these measurements are sensitive to interference patterns while the ejection of the solution and thinning of the wet film before crystallization. The pink dashed lines indicate the acceleration of the spin speed from 1000 rpm to 4000 rpm at 10 s within the process. The thinning of the following concentrations is presented: (a) 0.1 M, (b) 0.5 M, (c) 0.8 M, and (d) 1.2 M.

From the interference pattern, the wet-film thickness d is determined by:[16]

$$d = \frac{m}{2\sqrt{(n^2 + \sin^2\theta)}\Delta\nu}$$

For every spectrum in the time series, where m is the number of fringes per period in a spectral interval $\Delta\nu$ (in cm^{-1}), n is the refractive index, and θ is the angle of incidence of the white light. The solution refractive indices n are given in Table S3.

Table S3: Refractive index *n* for the 3CatPbBr$_3$ concentration series.

**Concentration / mol L^{-1}**	0.1	0.5	0.8	1.2
**Refractive Index**	1.450	1.473	1.489	1.511

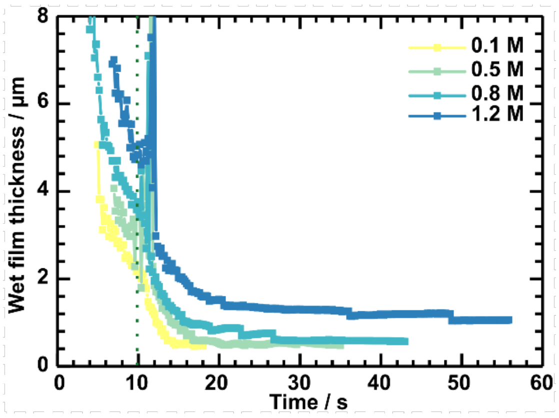

Figure S9: Fitted wet-film thinning for the established 3CatPbBr$_3$ concentration series. The green dotted line displays the time of spin speed acceleration from 1000 rpm to 4000 rpm.

Figure S9 presents the evolution of the wet-film thickness fitted from the interference patterns for the 0.1 M, 0.5 M, 0.8 M, and 1.2 M concentrations. In the first stage of spin-coating, flow dominates ejection and planarization of the solution thinning. Thinning is mainly executed by the solution flowing off at the sides of the substrate. As a result, the film thickness decreases abruptly for all concentrations. While acceleration, the interference pattern changes dramatically, resulting in a noisy fit for the wet film. Lower concentrated solutions thin faster. Hence, wet films prepared from the lower concentrated solutions are thinner. At later spin-coating times, the wet film thinning is mainly dominated by evaporation of the solvents, and thus the film thickness only decreases slowly. Overall, wet films spin-coated from higher concentrated solutions are thicker after finishing this process than lower concentrated solutions. For example, while the wet-film spin-coated from the 1.2 M solution has a thickness of ~1.3 µm, the wet-film prepared from the 0.5 M solution is ~0.5 µm thick. In total, the wet-film thinning is firmly concentration-dependent.

SI Note 3

Figure S11 (a) presents the wavelength-dependent absorption coefficient α of the solvent mixture 7:3 DMF:DMSO, the 0.1 M, and 0.5 M 3CatPbBr$_3$ solution. The pure solvents absorb below 250 nm and do not overlap with the absorption features of the precursor solutions above 280 nm. A redshift in the peak position and the absorbance onset for the 0.5 M 3CatPbBr3 solution indicates changes in the complex composition.

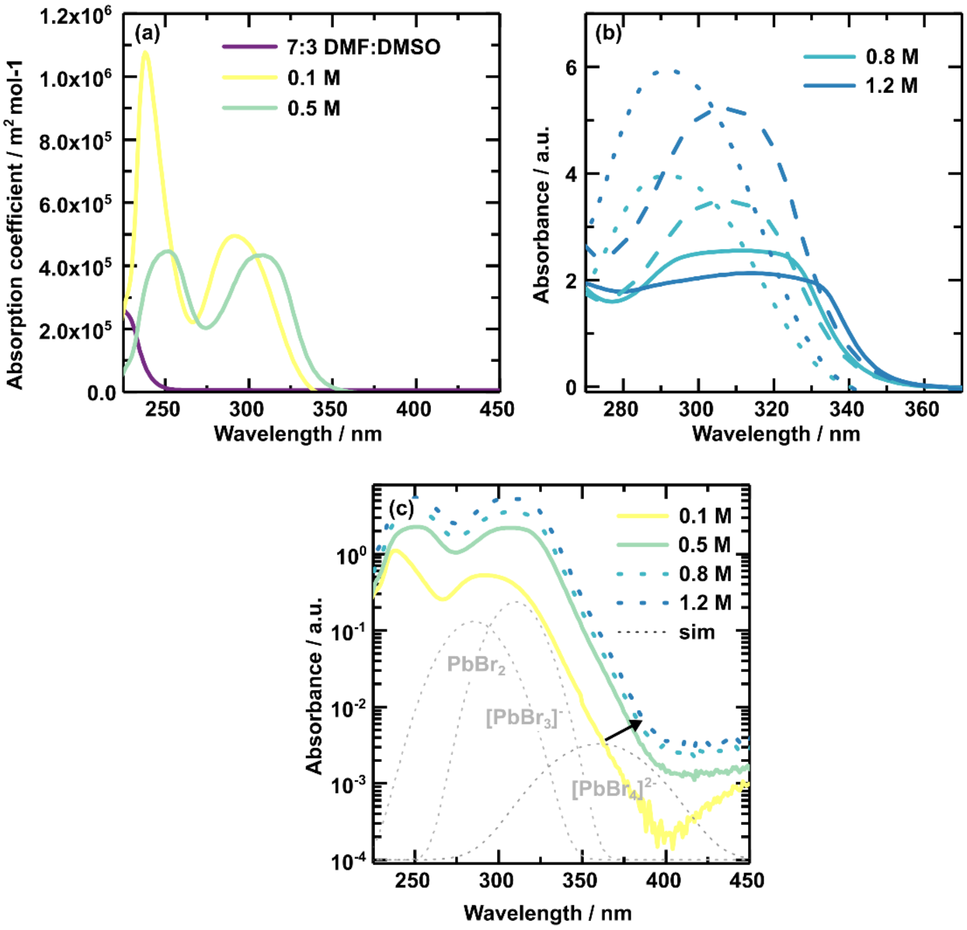

Figure S10: Concentration-dependent UV-vis data on the 3CatPbBr$_3$ solution series. The wavelength-dependent absorption coefficient (α) for the solvent system 7:3 DMF:DMSO, the 0.1 M, and the 0.5 M solution is presented in (a). Due to the overall high absorption coefficients, the higher concentrated solutions of 0.8 M and 1.2 M, shown in (b), saturated the detector (solid lines). Therefore, their absorbance is recalculated using α of the 0.1 M (dotted lines) and the 0.5 M solution (dashed lines), respectively. The absorbance for all four concentrations is plotted in (c), while solid lines present actual measurements and dashed lines recalculated absorbance spectra utilizing α of the 0.5 M solution. The grey dotted lines indicate solution species of MAPbBr$_3$ in pure DMF from Yoon *et al.*[17].

Perovskite solutions demonstrate high absorption coefficients. Thus, for the two higher concentrated solutions, no reliable absorbance spectra are detected (Figure S11 (b), solid lines) due to the oversaturation of the detector. Utilizing Lambert-Beer´s law, the absorbance spectra are recalculated. Assuming the absorption coefficient of the 0.1 M solution, an apparent discrepancy at the absorption onset is visible (dotted lines). With the absorption coefficient of the 0.5 M solution (dashed lines), the absorbance onsets for both measured and calculated spectra arise from 360 nm. Regardless, the rising onsets deviate slightly below 340 nm. Thus, the 0.5 M solution describes the chemical composition in the higher concentrations more accurately. Re-calculation accounts for intensity increases, not changes in the absorption coefficient's chemical composition, which evokes the discrepancies at the flanks. Shifts in the absorbance onset indicate concentration-dependent complex chemistry.

Figure S11 (c) presents the measured and recalculated absorbance on a logarithmic scale, more sensitive to slight absorbance increases below 400 nm. While $PbBr_2$ and $[PbBr_3]^-$ influence the absorbance around 285 nm and 310 nm, an increase in absorbance ~ 360 nm is attributed to $[PbBr_4]^{2-}$ (Figure S11 (c), grey dashed lines). Literature values are given for diluted solutions

of $MAPbBr_3$ in pure DMF.[17] Exact absorbance positions will change due to composition and solvent system, e.g., by solvatochromic effects. Hence, solution-based UV-vis measurements indicate a shift in the chemical equilibrium to higher coordinated lead-bromide complexes of poly-complexes forming for higher concentrated solutions.

Literature:

[1] M. Kulbak, I. Levine, E. Barak-Kulbak, S. Gupta, A. Zohar, I. Balberg, G. Hodes, D. Cahen, *Adv. Energy Mater.* **2018**, DOI 10.1002/aenm.201800398.

[2] Y. Rakita, N. Kedem, S. Gupta, A. Sadhanala, V. Kalchenko, M. L. Böhm, M. Kulbak, R. H. Friend, D. Cahen, G. Hodes, *Cryst. Growth Des.* **2016**, *16*, 5717–5725.

[3] M. Saliba, J. P. Correa-Baena, C. M. Wolff, M. Stolterfoht, N. Phung, S. Albrecht, D. Neher, A. Abate, *Chem. Mater.* **2018**, *30*, 4193–4201.

[4] P. Ferdowsi, E. Ochoa-Martinez, U. Steiner, M. Saliba, *Chem. Mater.* **2021**, *33*, 3971–3979.

[5] A. Merdasa, C. Rehermann, K. Hirselandt, J. Li, O. Maus, F. Mathies, T. Unold, J. Dagar, R. Munir, E. L. Unger, **2020**, DOI 10.21203/RS.3.RS-102041/V1.

[6] M. Krumrey, G. Ulm, *Nucl. Instruments Methods Phys. Res. Sect. A Accel. Spectrometers, Detect. Assoc. Equip.* **2001**, *467*, 1175–1178.

[7] A. Hoell, I. Zizak, H. Bieder, L. Mokrani, *DE102006029449* **2007**.

[8] U. Keiderling, *Appl. Phys. A* **2002**, *74*, s1455–s1457.

[9] I. Breßler, J. Kohlbrecher, A. F. Thünemann, *J. Appl. Crystallogr.* **2015**, *48*, 1587–1598.

[10] D. Frenkel, S. Auer, *Nature* **2001**, *409*, 1020–1023.

[11] D. Orsi, A. Fluerasu, A. Moussaïd, F. Zontone, L. Cristofolini, A. Madsen, *Phys. Rev. E 85* **2012**, *85*, 011402.

[12] A. Vrij, J. W. Jansen, J. K. G. Dhont, C. Pathmamanoharan, M. M. Kops-Werkhoven, H. M. Fijnaut, *Faraday Discuss. Chem. Soc.* **1983**, *76*, 19–35.

[13] A. Vrij, *J. Chem. Phys.* **1979**, *3267*, 3267–3270.

[14] J. K. Percus, G. J. Yevick, *Phys. Rev.* **1958**, *110*, 1–13.

[15] J. Mooney, P. Kambhampati, *J. Phys. Chem. Lett.* **2013**, *4*, 3316–3318.

[16] N. J. Harrick, *Appl. Opt.* **1971**, *10*, 2344.

[17] S. J. Yoon, K. G. Stamplecoskie, P. V. Kamat, *J. Phys. Chem. Lett.* **2016**, *7*, 1368–1373.
